# Characteristics of the Manifestation of Multiple Sclerosis in Children in Lithuania

**DOI:** 10.3390/medicina59061055

**Published:** 2023-05-30

**Authors:** Brigita Afanasjeva, Dominykas Afanasjevas, Milda Endzinienė, Renata Balnytė

**Affiliations:** 1 Department of Neurology, Hospital of Lithuanian University of Health Sciences Kauno Klinikos, 50161 Kaunas, Lithuania; 2 Department of Neurology, Faculty of Medicine, Lithuanian University of Health Sciences, 50161 Kaunas, Lithuania; 3 Department of Maxillofacial Surgery, Faculty of Medicine, Lithuanian University of Health Sciences, 50161 Kaunas, Lithuania

**Keywords:** multiple sclerosis, children, initial symptoms, radiological findings, relapse, disability

## Abstract

*Background and Objectives*: Multiple sclerosis (MS) starts quite rarely in childhood, comprising just 3–10% of all diagnosed cases of MS population. The age of onset of the disease may be related to the initial phenotype and the prognosis of MS. The aim of the study is to assess the characteristics of the manifestation of MS in children. *Materials and Methods*: Two groups of patients were analyzed: those diagnosed with MS in childhood (0 < 18 years of age) and who developed MS in 2005–2021, and those diagnosed in adulthood (≥18 years old). The data were collected from the database of the Lithuanian University of Health Sciences Kauno Klinikos. *Results*: For the analysis, 105 patients were selected: 35 children (group A) and 70 adults (group B). At the onset of the disease, 62.9% of children and 70.0% of adults experienced visual disturbances (*p* > 0.05). Isolated symptoms were more common in children (65.7%) as compared to adults (28.6%), *p* < 0.001. Sensory disorders were more common in adults than in children (*p* < 0.001). Optic nerve and cerebral hemispheres were the most affected in group A (*p* < 0.05). During the first year after diagnosis, the median number of relapses in group A was higher (3, range 1–5) as compared to group B (1, range 1–2) (*p* < 0.001). Recovery time after a relapse was shorter in children as compared to adults (*p* < 0.001). Oligoclonal bands were found in 85.7% of children and in 98.6% of adults. Oligoclonal bands were less common in the childhood-onset than in the adult-onset group (*p* = 0.007). *Conclusions*: The initial symptoms of multiple sclerosis in pediatric patients usually appeared around the age of 16, with a similar frequency in boys and girls, and in most of the childhood cases the initial symptoms were limited to the dysfunction of a single part of the nervous system children usually started with visual disorders, while sensory, coordination and motor disorders were less common. The course of the disease in juvenile patients with MS was more aggressive in the first year as there were more relapses, but the functional impairment recovered faster as compared to adults.

## 1. Introduction

Multiple sclerosis (MS) is a chronic inflammatory autoimmune disease that manifests because of progressive inflammatory and demyelinating processes in the central nervous system, usually starting at a young age, leading to disability of various degrees and thus creating an important health problem in neurology. An increase in MS incidence rates was observed in a nationwide study in Lithuania by using the data spanning from 2001 to 2015 by using the Compulsory Health Insurance Information System. The data showed a surge of yearly MS diagnosis, with the tendency to increase every year during the investigated period from 5.7/100,000 in 2001 to 8.1/100,000 population in 2015, with females being 1.5–2 times more likely to develop MS compared to males [1,2,3]. MS starts on average between 28 and 31 years of age, but an earlier onset before 18 years is also possible, comprising 3–10% of all diagnosed MS cases. The reported incidence of MS in the childhood population is 0.66–1.66/100,000 new cases per year. The age of onset of the disease may be related to the phenotype and the prognosis of MS [1,2]. The initial symptoms and the course MS are thought to be slightly different in childhood and adult populations, though scientific data regarding this issue are quite scarce.

The question arises why such differences exist between children and adults with multiple sclerosis. According to the literature, it is possible due to differences in the white matter microstructure between children and adults: a more mature white matter microstructure in adults compared to children may be associated with a later onset of the disease. Brain plasticity is characteristic of children with multiple sclerosis, which could explain the faster and better recovery in children after relapses. It is possible that immunoregulatory mechanisms lead to better recovery in children with multiple sclerosis, and the latter are more likely to eliminate malignant cells and restore homeostasis at a younger age. In addition, the MRI images show more active lesions in children than in adults, as a result of more active immune system of children. There is an increased proliferation of myelin peptides and T cells in patients with childhood disease compared to adults, and this leads to different immune system responses and different disease characteristics (recovery after relapses, severity of disability) between children and adults. According to studies, the expression as well as age and timing of first symptoms, diagnosis and treatment of multiple sclerosis may be important for the phenotype and prognosis of multiple sclerosis [4,5].

There is a lack of data and research on multiple sclerosis in children in Lithuania. Furthermore, this is the first research of its kind in Lithuania. The aim of this study was to determine the characteristics of MS in patients who developed MS in childhood and to compare these characteristics with the ones found in adult-onset population in Lithuania.

## 2. Materials and Methods

This retrospective case-control study was conducted at the Neurology Department, Hospital of Lithuanian University of Health Sciences Kauno Klinikos (KK). The consent of the Lithuanian Bioethics Committee No. 2023-BE10-0001, 23 January 2023, was obtained for the study. The patients included in the study were divided into two groups: group A consisted of patients diagnosed with MS in childhood (0 ≤ 18 years of age) and group B those diagnosed with MS in adulthood (≥18 years of age), based on McDonald’s criteria in both groups. Candidates for group A (patients diagnosed with MS before age 18 and within the period 2005–2021) were selected from the patient database and other relevant medical documents available at KK, and the candidates for the control group (B) were randomly selected from the same sources, regardless of dates of their disease onset. Juvenile patients with other demyelinating diseases such as acute disseminated encephalomyelitis (ADEM) were not included in the study.

The following data were collected for both groups: gender; age; the initial symptoms of the disease (later divided into the following groups: visual, motor, sensory, dysfunction of pelvic organs, fatigue, coordination disorders, cognitive dysfunction, combined symptoms (>1 clinical symptom)); age at MS diagnosis; disability (at diagnosis and after first relapse assessed by using the Kurtzke Expanded Disability Status Scale (EDSS)); number of relapses within one year of the diagnosis; time interval between the first and the second relapse (in days); presence of oligoclonal bands in the cerebrospinal fluid (CSF; investigation performed at time of diagnosis); characteristics of the brain magnetic resonance imaging (MRI) findings (the type of the lesions and the activity of the foci found on the first imaging as well as new foci during the course of the disease within the period of one year); and location of the lesions at the time of diagnosis (dividing the findings into the following groups: optic nerve, brainstem, brain hemispheres, cerebellum, spinal cord, and multiple parts of the nervous system).

The clinical data of group A were compared with the data of the control group (B). Statistical data analysis was performed by using the statistical program package IBM SPSS 27.0.1. Descriptive numerical characteristics were calculated: number of cases and frequency in percent, median, and 25–75 percentiles (Q1–Q3). The Shapiro–Wilk test was used to check the data distribution. For the comparison of independent samples, in case of non-normal distribution of cases, the Mann–Whitney U test was applied. Data were considered statistically reliable when *p* < 0.05.

## 3. Results

For the study, 105 patients were selected: 35 (female 18 (51.4), male 17 (48.6)) children (group A) and 70 (female 40 (57.1), male 30 (42.9)) adults (group B). The male/female ratio did not differ significantly between the groups, *p* = 0.68. The median age (Q1–Q3) in group A was 16 (14–17) years and in the control (group Q1–Q3) was 36 (29–44) years.

At the onset of the disease, 62.9% of children and 70.0% of adults experienced visual disturbances (*p* > 0.05). Sensory disorders at the onset of the disease were more common in adults with MS than in children (*p* < 0.001). A total of 12 (34.3%) children had symptoms of impairment to more than one area of the central nervous system (combined symptoms) at the onset of the disease, and 23 (65.7%) children had isolated symptoms. In the control group (B), 50 (71.4%) subjects had more than one system of neurological disorder (combined symptoms) at the disease onset, and 20 (28.6%) subjects had isolated symptoms. Combined symptoms were significantly more common in adults than in children (*p* < 0.001) (Table 1).

Table 2 shows the localization of the first lesion detected on MRI. Optic nerve and cerebral hemispheres were the most affected sites in the childhood-onset group (A), and these sites were more commonly affected in group A as compared to group B (*p* < 0.05). The majority of MRI scans of the adult-onset patients (64.3%) showed multiple sites of lesions, which was less characteristic for the childhood-onset group (28.6%) (*p* < 0.001).

New active foci on MRI were detected in 19 (54.3%) and new inactive foci in 16 (45.7%) cases with childhood-onset MS. In the control group (B), 55 (78.6%) of the cases were diagnosed with new active foci and 15 (21.4%) with new inactive foci. In the childhood-onset group (A), new active foci were significantly less common and inactive foci significantly more common as compared to the adult-onset group (*p* = 0.01).

In group A, 34 (97.1%) patients had a relapsing–remitting form of MS, and 1 (2.9%) patient had a secondary progressive form. In group B, 67 (95.7%) of the subjects had a relapsing–remitting form of MS, and 3 (4.3%) subjects had a primary progressive form (*p* = 0.17). The number of patients with the relapsing–remitting form of MS did not show any differences between the groups (*p* = 0.17).

During the first year after diagnosis, the median number of relapses (Q1–Q3) in group A was higher (3, range 1–5) as compared to group B (1, range 1–2) (*p* < 0.001). The degree of disability according to the EDSS was distributed as follows: in the childhood-onset group (A), the median degree of disability (Q1–Q3) at the onset of a relapse reached 2.0 points (1.5–3.0), and after the relapse reached 1.5 (1.0–2.0). In the adult group, the degree of disability was 2.5 (2.0–3.5) at the onset of the relapse and 2.0 (1.5–2.5) after clinical remission was achieved. Median recovery timeframe after the relapse (Q1–Q3) was 4 (3–6) days in the childhood-onset group and 7 (5–10) days in the adult-onset group. Recovery time after a relapse was shorter in children as compared to adults (*p* < 0.001).

Oligoclonal bands were found in 30 (85.7%) children and in 69 (98.6%) adults, and oligoclonal bands were less common in the childhood-onset than in the adult-onset group (*p* = 0.007).

## 4. Discussion

In our study, we found that the onset of multiple sclerosis in children was between 14 and 17 (median 16) years of age, which matches with the literature data [6,7]. Some studies report that the majority of juvenile patients with MS are girls, especially those older than 10 years, and it is considered that the onset of the disease might be influenced by hormonal changes during puberty [1,7,8,9]; however, our study did not show any gender differences in either the childhood or adult-onset groups.

We found that the onset of MS in children started as an isolated symptom significantly more often than in adults (65.7 and 28.6 percent, respectively). This differs from some literature sources which state that MS in children usually manifests not as a single symptom but rather a combination of symptoms. On the other hand, there are literature data showing that in adolescence MS often presents with an isolated symptom similar to adults, and in our study the majority of the population of children were adolescents [1,5,10,11,12].

In children, the most common initial MS symptom was vision disorder, while motor, sensory and coordination disorders occurred less often. In adults, the most common symptom was visual disorder as well, followed by motor and sensory disorders, which occurred with similar frequency; thus, our results matched the literature data [1,13,14,15]. Our study found that children less often experienced sensory disorders as compared to adults, and this also matches the literature data [5]. This finding might be explained by the difficulties for children to identify and verbalize their sensory impairments, or they may not have been interviewed well enough about the sensory impairments, and some authors suggests that the diagnosis of MS may be delayed in children because they may be unable to identify their sensory impairments [11,16]. There may also be other symptoms that children may not express, such as dysphagia [17]. According to the literature, one third of children with MS may have cognitive function disorders, and the cognitive areas most commonly affected in children with MS are attention, memory, speed of information integration, language and spatial and visual integration, but during our performance study, this was not examined [1,18].

In our study, we found that the prevailing localization of the MRI lesions in the childhood-onset group was optic nerve and cerebral hemispheres. In the adult-onset group, these two sites of lesion were also common, though not as much as in the childhood-onset group. Isolated lesions were more common in children than in adults who quite often (64.3%) were diagnosed with multiple lesions at different locations on their first MRI. We may assume that there is a danger that an isolated MRI lesion may be underlooked in children who in addition may not be able to clinically verbalize some of their sensation disorders, thus resulting in the diagnostic delay. According to the literature, lesions can usually be found in various parts of the brain for children and adults. [1,4].

We found that the relapsing–remitting form of MS is most common in children. Some authors suggest that the relapsing–remitting form of MS is usually diagnosed in children because they are diagnosed relatively early, and it takes more time for the secondary progressive form of the disease to develop as compared to adult-onset MS patients [6,10,13]. In our study, the diagnosis of the relapsing–remitting disease form was equally common often in both groups.

We found that juvenile patients had a higher number of relapses within a year of diagnosis as compared to adults, and the recovery time was shorter in children than in adults. These differences are also described in the literature [4,10]. It is believed that this may be due to the greater plasticity of the children’s brain, and it is also possible that better recovery in the childhood-onset group of patients is due to immunoregulatory mechanisms, which at a younger age are more efficient at the removal of malignant cells and the restoration of homeostasis [4,10]. According to the literature, children had higher disability scores at the start of a relapse and a significant decrease in the scores by the end of the relapse as compared to adults [1,4,10,14]. However, according to the data of our study, the disability at the start of the first relapse was higher in the adult group, and after the relapse the disability scores decreased by 0.5 points in both groups.

According to the literature, more active foci were found at initial MRI in children than in adults, and it is considered that this might be related to the fact that children have a greater inflammatory response than adults [12,15]. However, in our study the results were the opposite—the children had significantly fewer new active foci, and mostly inactive foci were identified as compared to the control group of adults. We may speculate that this prevalence of inactive foci might be related to the isolated occurrence of clinical symptoms in our children. Some authors report higher total volume of the lesion in children than in adults; unfortunately, we could not evaluate the lesion volume in our study because of its retrospective design, although this might be important and therefore is recommended to be investigated in future studies. Most authors have reported that lesions on MRI images in children were larger (in terms of lesion volume, active and new foci) than in adults, and their recovery was better than in adults, but in the long term, children with multiple sclerosis had a higher degree of disability than adults, and this might be because of the disability accumulation factor [1,14,15].

According to other authors, the finding of oligoclonal bands in the cerebrospinal fluid was found to be lower in children under 10 years of age than in adults [6]; the data of our study are analogous, although the children in our study were of various ages, with a median age of 16 years. The question why oligoclonal bands are less common in children than in adults is difficult to answer, possibly because of the greater immune response in children [6,16].

Advantages of our study included a long period of time during which MS patients developed in childhood was examined (2005 and 2021) and the case-control type of the study design which allowed comparisons between the childhood-onset and the adult-onset patient populations, and clinically significant differences were found. The conducted research also has shortcomings: it is a retrospective study, and therefore the data were collected from medical documentation and the descriptions of some data may lack the accuracy required for a precise scientific study; in the study of radiological images, only the foci and their activity were analyzed, not the volume of the lesion; the course and the progression of the disease was not observed in a long-term manner. The study did not examine other diseases or the EBV status of the patients, which could have influenced the course of the disease. During the study, it was not possible to evaluate and compare the duration of the disease in both groups.

## 5. Conclusions

The initial symptoms of multiple sclerosis in pediatric patients usually appeared around the age of 16, with a similar frequency in boys and girls. Unlike adults, in most of the childhood cases, the initial symptoms were limited to the dysfunction of a single part of the nervous system, while combinations of symptoms manifested more often in adults than in children. Similar to adults, children usually started with visual disorders, while sensory, coordination and motor disorders were less common. Sensory impairment occurred significantly less frequently in children than in adults. The course of the disease in juvenile patients with MS was more aggressive in the first year as there were more relapses, but the functional impairment recovered faster as compared to adults.

## Figures and Tables

**Table 1:** Prevalence of initial symptoms of multiple sclerosis in childhood-onset and adult-onset populations.

Number of Patients with Clinical Symptoms	Patient Groups		
	A, N (%)	B, N (%)	*p*
Visual	22 (62.9)	49 (70.0)	0.461
Motor	12 (34.3)	34 (48.6)	0.164
Sensory	10 (28.6)	39 (55.7)	0.009
Pelvic dysfunction	0	4 (5.7)	0.149
Fatigue	0	4 (5.7)	0.149
Dyscoordination	9 (25.7)	28 (40.0)	0.149
Cognitive dysfunction	0	3 (4.3)	0.214
Combined symptoms	12 (34.3)	50 (71.4)	0.001

Abbreviations: A: patients with onset of multiple sclerosis in childhood; B: patients with onset of multiple sclerosis in adulthood.

**Table 2:** Localization of demyelinating foci in both patient groups on the magnetic resonance imaging.

Number of Cases with a Specific Localization of Demyelinating Focus	Patient Groups		
	A, N (%)	B, N (%)	*p*
Optic nerve	10 (28.6)	8 (11.4)	0.03
Brainstem	3 (8.6)	4 (5.7)	0.58
Cerebral hemispheres	9 (25.7)	7 (10)	0.04
Cerebellum	3 (8.5)	3 (4.3)	0.37
Spinal cord	0	2 (2.9)	0.313
Multiple localization	10 (28.6)	45 (64.3)	<0.001

Abbreviations: A: patients with onset of multiple sclerosis in childhood; B: patients with onset of multiple sclerosis in adulthood.

## Data Availability

Not applicable.
